# The Association of Vitamin D, Nerve Growth Factor (NGF), Brain-Derived Neurotrophic Factor (BDNF), and Glial Cell-Derived Neurotrophic Factor (GDNF) with Development in Children

**DOI:** 10.3390/children12010060

**Published:** 2025-01-06

**Authors:** Mia Milanti Dewi, Akhmad Imron, Nelly Amalia Risan, Grace Mediana, Raden Tina Dewi Judistiani, Budi Setiabudiawan

**Affiliations:** 1 Child Health Department, Faculty of Medicine, Universitas Padjadjaran, Hasan Sadikin Hospital, Bandung 40161, West Java, Indonesia; nelly.amalia@unpad.ac.id (N.A.R.); budi.setiabudiawan@unpad.ac.id (B.S.); 2 Neurosurgery Department, Faculty of Medicine, Universitas Padjadjaran, Hasan Sadikin Hospital, Bandung 40161, West Java, Indonesia; a.imron@unpad.ac.id; 3 Bandung City Health Service, Bandung 40161, West Java, Indonesia; gracemediana1412@gmail.com; 4 Public Health Department, Faculty of Medicine, Universitas Padjadjaran, Bandung 40161, West Java, Indonesia; tina.d.judistiani@unpad.ac.id

**Keywords:** child development, glial cell, neurotrophic factors, nerve growth factor, vitamin D

## Abstract

Background: Short stature remains a global problem and is associated with vitamin D status. Vitamin D is also a neurosteroid with regard to neurotrophic factors but its role in development is unclear. Therefore, this study analyzed the relationships between vitamin D, NGF, GDNF, and BDNF and developmental status in children with a history of short stature (<2 years). Methods: This research is a prospective cross-sectional study conducted in March 2022. The vitamin D, NGF, GDNF, and BDNF levels were measured in stored biological materials from children aged 2–4 years, and their Ages and Stages Questionnaire (ASQ-3) scores were also assessed. The results were analyzed via the chi-square test, Fisher’s exact test, Mann–Whitney test for NGF, unpaired *t*-test, and Spearman rank correlation. Results: Among the 85 study subjects, 41.2% were short in stature, with 37% having developmental deviation. Male sex (*p* = 0.038) and low maternal education (*p* = 0.024) were associated with short stature. The mean vitamin D level was lower (*p* = 0.041) in children with short stature (27.65 ng/dL). The risk factors associated with short stature were vitamin D levels ≤ 32.7 ng/dL, GDNF levels ≤ 12.99 ng/mL, male sex, and low maternal education. Children with short stature (<2 years old) also demonstrated impaired problem-solving as assessed by the ASQ-3 (*p* = 0.005). Vitamin D was also associated with gross motor skills (*p* = 0.035) and personal social development (*p* = 0.038). Conclusions: There was no association of vitamin D with NGF, GDNF, or BDNF levels. Vitamin D levels are associated with short stature and development in children, especially gross motor skills, personal social development, and problem solving.

## 1. Introduction

Short stature based on the World Health Organization (WHO) Child Growth Standards curve remains a problem around the world. Over half of the world’s stunted children under five years old (roughly 55%) are from Asian nations. In Southeast Asia, Indonesia has the third-highest rate of stunting, according to the WHO. In 2016, 22.9% of children under 5 years of age had short stature globally [1,2,3]. Based on data from the 2018 Indonesian Health Profile, the rate of short stature in children aged 0–23 months was 29.9%, and aged 0–59 months, it was 30.8% [4]. Children’s development and growth accelerate throughout the first 1000 days of life. During this period, micronutrient deficiencies, such as those in vitamin D, might result in short stature. Approximately 30% of short stature cases in Indonesia are thought to be related to vitamin D deficiency [1,5,6]. Short stature can impact growth, but it can also raise the risk of degenerative diseases and have long-term impacts on cognitive function. Children with short stature are 3.71 times more likely to experience developmental deviations in gross motor, fine motor, language, and personal social domains [7]. Therefore, screening for such deviations should be performed from an early age via highly sensitive and specific tools such as the ASQ-3 (sensitivity of 88% and specificity of 94%) [8,9].

Brain development is regulated by various factors, including neurotrophic factors. Vitamin D functions as a neurosteroid for several neurotrophic factors, including nerve growth factor (NGF), brain-derived neurotrophic factor (BDNF), and glial cell-derived neurotrophic factor (GDNF). The relationship between neurotrophic factors and brain disorders has been widely studied in adults, but few studies have been conducted in children [10,11,12,13]. Vitamin D deficiency has become a global problem, with children being at high risk of vitamin D deficiency [14,15,16,17]. Even in nations with adequate exposure to sunlight, vitamin D insufficiency is common. About 70% of Asians suffer from vitamin D deficiency, with Southeast Asia having a prevalence of 6–70%, Indonesia having a prevalence of >50%, and Malaysia having a prevalence of 47–75% [18]. Animal and in vitro studies have shown that vitamin D affects brain development and that children with vitamin D deficiency experience developmental abnormalities [14,17,19,20]. Currently, there is no research regarding the relationships among vitamin D, neurotrophic factors, and development in children with short stature; therefore, this study was designed to investigate this relationship to help overcome the problem of child development and short stature in Indonesia.

## 2. Materials and Methods

This study is a prospective cross-sectional study, conducted in March 2022, to assess the role of vitamin D in the relationship of NGF, GDNF, and BDNF with the developmental status of children <2 years old with a history of short stature. The study population consisted of 85 children, with the following inclusion criteria: children aged <2 years, participation in the ALG Grant research entitled ’The Role of Vitamin D in Reducing Maternal and Infant Mortality Rates throughout Bandung Regency’, parents having completed at least elementary school, and parents having consented to participate in the study. Children with a history of premature birth, low birth weight, numerous anomalies, syndromic conditions, and non-spontaneous birth were excluded from this study. The samples used in this study were taken via a simple random sampling method in the target population.

The subjects had their height assessed and were considered short stature if height was < −2SD according to the WHO CGS curve by sex. Then, blood samples were taken and sent to the clinical pathology laboratory of Dr. Hasan Sadikin Hospital, Bandung. The examination material was serum-stored chemicals derived from blood samples that had been taken. Before the examination of vitamin 25 (OHD), NGF, GDNF, and BDNF, serum was stored at −80 °C. Research materials and reagents were processed using an ELISA examination reagent kit (Euroimmune R, Lübeck, Germany). The researcher then conducted a developmental examination with the ASQ-3 according to age category, and the results were then analyzed.

The research data were analyzed univariably to describe the basic characteristics of the subject and the research variables. Numerical data are presented in the form of means, medians, standard deviations, and maximum minimum ranges. The categorical data are presented as percentage or proportion. Furthermore, bivariable analysis was carried out to connect the independent variable with the dependent variable. Since all variables for bivariable analysis were numerical variables, the bivariable analysis used in this study was the Pearson correlation test or simple linear regression. This bivariable analysis also included a selection of variables that became multivariable candidates. The variables that were entered into the multivariable model are those variables that in the bivariable analysis had a *p* value of 0.25. Finally, multivariable analysis was performed via multivariable linear regression. The assumptions that must be met in the multiple linear regression analysis were as follows: the final result of linear regression analysis is a model equation that can predict the incidence of short stature; beta values generated from the output of multivariate analysis can be used to determine which variables play a major role in the occurrence of short stature; the greater the beta value, the greater the influence on the short stature variable.

## 3. Results

Among the children in Bandung Regency who were included in the research entitled ’The Role of Vitamin D in Reducing Maternal and Infant Mortality Rates throughout Bandung Regency’ who met the inclusion criteria, 1 subject died, 6 subjects could not be examined, and 15 subjects moved home; thus, the data from 85 children were analyzed in this study. The children’s characteristics are summarized in Table 1. Thirty-five of the 85 subjects were of short stature, and 13 (37%) had developmental deviations; of the 50 children of normal stature, 15 (30%) had developmental deviations. Male sex (*p* = 0.038) and low maternal education (*p* = 0.024) were associated with short stature.

The multivariate analysis of factors associated with short stature in children <2 years old is presented in Table 2. The statistical analysis revealed that maternal education was ≥high school education, and the OR increased with the inclusion of gender and education. The R2 of 0.392 means that 39.2% of short stature cases <2 years old are influenced by low vitamin D and GDNF levels, gender, and the mother’s education.

Table 3 compares the vitamin D, NGF, GDNF, and BNDF levels in the short-stature group with those in the normal-stature groups, revealing that the vitamin D levels were lower in children with short stature (*p* = 0.041). The children’s development was evaluated via the ASQ-3, which assesses communication, gross motor and fine motor skills, problem solving, and personal social development. The results revealed that the children with short stature experienced significant disturbances in problem solving (*p* = 0.005).

Table 4 shows that there was no relationship between the biomarkers measured and developmental status according to the ASQ-3 in children aged 2–4 years and that the average levels of NGF, GDNF, and BDNF were not significantly different between the abnormal development groups with short stature and those with normal stature. Communication in the children as assessed by the ASQ-3 was not significantly affected by vitamin D or biomarker levels. However, vitamin D levels are correlated with gross motor skills (*p* = 0.035) but not fine motor skills in children with a history of short stature aged < 2 years. There was a significant relationship between vitamin D and personal social development (*p* = 0.035) in children with a history of short stature aged < 2 years and between the abnormal development groups with short stature and normal stature.

Regarding the relationship between vitamin D and the various biomarkers, only the levels of GDNF and BDNF appear to be correlated in children with short and normal stature (Table 5).

The cutoff levels of vitamin D and the various biomarkers for the prediction of short stature are shown in Table 6. Accordingly, children aged <2 years who have vitamin D levels ≤ 32.7 ng/dL have a risk of short stature 4.83 times greater than children with vitamin D levels > 32.7 ng/dL (Table 6).

## 4. Discussion

Approximately 40% of our study cohort of Indonesian children aged <2 years was classified as having short stature according to the WHO criteria. This is in accordance with a study from Karlsson et al. (2022), which found that the prevalence of stunting was 32% and that older children are more likely to have stunting until they are about 28 months old [21]. Levels were lower in a study from Laksono et al. (2022) that reported that 20.1% of Indonesia’s under-two-year-old population is stunted [22]. In Indonesia, the prevalence of short stature is decreasing annually in line with WHO targets to reduce the rate of short stature by 40% in children under 5 years of age by 2025 [1].

Vitamin D is a fat-soluble vitamin that is important for skeletal function as well as various metabolic processes. Levels of 25-hydroxy vitamin D (25(OH)D3 of 21–29 ng/mL are categorized as insufficient. [18,23,24]. Vitamin D deficiency has become a global problem. Various studies mention the prevalence of vitamin D deficiency in Asia of around 70%, Southeast Asia 6–70%, and Malaysia 45–75% [18,23]. The mean vitamin D level determined by measuring D (25(OH)D3) in short-stature subjects was 27.65 ng/mL (10.5–39.8 ng/mL). This aligns with a study from Octavius et al. (2023) that reported a mean serum vitamin D level of 22.74 ng/mL (95% CI, 16.95–30.51) [25]. Levels were lower in a study from Ganmaa et al. (2024), with a mean serum vitamin D level of 11.9 ng/mL (4.2) [26].

Vitamin D levels were significantly related to body length; the lower the vitamin D level was, the shorter the body length was. The multivariable analysis revealed that several variables were related to short stature, namely vitamin D and GDNF levels, as well as male sex and low maternal education. The body length of male children was significantly shorter than that of female children in our study cohort. Typically, boys grow slower than girls but experience rapid growth during puberty. Growth monitoring is necessary every 6–12 months, with a decrease in the growth rate to lower than the first percentile indicative of a growth disorder [27].

Most mothers in the short and normal groups had an elementary school or junior high school education, and this was statistically significant. Maternal education is very important in parenting, as mothers provide nutrition and monitor their child’s growth and development. Laksono et al. (2022) reported that a mother with a primary school education had a risk of having a stunted child of 1587 times greater than average, whereas a mother educated at junior high school had a risk of stunting that was 1430 times greater [22]. Some mothers also do not work, with family incomes below the regional minimum wage (UMR), which in the Bandung Regency in 2023 is IDR 1,986,670.00. Additionally, the COVID-19 pandemic has significantly impacted families’ incomes.

Research in Bangladesh shows that there is no difference in vitamin D levels in children with short stature, whereas Mokhtar’s study (2017) reported that vitamin D is associated with short stature aged 6–36 months in Ecuador [19,28]. Moreover, in India, vitamin D supplementation increased the growth of short-statured children [29,30].

Low vitamin D levels are associated with a lack of sun exposure as well as a low intake of food containing vitamin D or disturbed vitamin D metabolism. Low vitamin D intake can be influenced by the mothers’ lack of education and low socioeconomic status, as in this study. Children should eat foods rich in vitamin D, such as nuts, fish, milk, eggs, meat, or fruit, to prevent growth disorders [31]. Exposure to sunlight for 30 min/week for babies in diapers and 2 h/week for babies wearing full clothes without a hat can maintain vitamin D levels > 11 ng/dL [31]. Research in Saudi Arabia recommends increasing outdoor physical activity to increase sun exposure, and children in Poland are recommended to have at least 15 min of sun exposure between 10:00 and 15:00 [32].

Vitamin D in a child’s developing brain influences neurotrophic function, neuroprotection, and structural development [14]. Neurotrophic function plays a role in neurotransmitter synthesis, nerve cell differentiation, reducing apoptosis in the hippocampus, regulating memory receptors, and regulating nerve growth factors. Neuroprotective function and structural development influence anti-inflammatory effects in the brain, the regulation of nitrite and oxygen related to apoptosis, the regulation of intracerebral detoxification, language development, and the repair of damaged DNA [14]. Vitamin D in the brain functions as a neurosteroid for neurotrophic factors such as NGF, GDNF, and BDNF. NGF plays a role in cholinergic nerves related to behavior in the forebrain cortex, hippocampus, thalamus, brainstem, and dorsal root ganglia. Cholinergic nerve cells mostly use acetylcholine as a neurotransmitter to send messages. GDNF strengthens dopaminergic nerve cells in the midbrain area, which is associated with gross motor movements. BDNF plays a role in dopaminergic nerves in the hippocampus, amygdala, cortex, hypothalamus, and septum areas, which are related to speech/language processes [33,34,35]. The dopaminergic system uses dopamine as a neurotransmitter when messages related to motor skills, behavior, intelligence, and emotions are sent. In vitamin D deficiency, the mechanism by which neurotrophic factors maintain cell life, differentiation, and synaptic plasticity is thought to be reduced, which can cause cell death [33,36].

NGF is involved in regulating the central and peripheral nervous systems, which play important roles in postnatal development [37]. Vitamin D plays a role in NGF circulation and prevents increased calcium caused by β-amyloid. Different amyloids also suppress vitamin D receptor expression. Furthermore, experimental animals given vitamin D have shown increased NGF levels [38]. However, NGF was not influenced by vitamin D levels and had no significant effect on the child’s developmental status in the present study.

It has been reported that giving vitamin D could increase dopamine levels but not BDNF and serotonin levels in ADHD children [35]. Bilgic et al. also reported changes in GDNF but not NGF and BDNF in ADHD patients, whereas Guney et al. reported increased NGF in ADHD patients, and Yeom et al. reported that BDNF plays a role in the behavior and intelligence of preschool children [12,34,39]. Budni et al. suspected changes in NGF, GDNF, and BDNF in elderly people and Alzheimer’s patients [40]. Studies in mice have shown that vitamin D regulates NGF [38].

This study also determined the cutoff values of vitamin D, NGF, GDNF, and BDNF in the prediction of short stature. The normal levels of these three neurotrophic factors have not been determined because the research has been limited to experimental animals. The cutoff values were ≤32.7 ng/mL for vitamin D, >1315.5 ng/mL for NGF, ≤12.99 ng/mL for GDNF, and ≤1803.6 ng/mL for BDNF. This is lower than findings from a study from Mokhtar et al. (2018), where the mean concentration of 25(OH)D in serum was 58.0 (SD 17.7) nmol/L. An undernutrition-specific 25(OH)D cutoff of <42.5 nmol/L was found by sensitivity analysis; serum 25(OH)D was less than 42.5 nmol/L in 18.6% of children. Stunted children were more likely to have low serum 25(OH)D levels (aOR = 2.8; 95% CI 1.6, 4.7) [28]. To the best of our knowledge, this is the first study to evaluate the NGF, GDNF, and BDNF cutoff values. There is hence very little comparison of the parameter value to predict stunting.

Children’s development should be monitored from birth, especially up to the first 1000 days of life. Development is influenced by many factors, especially nutrition (macronutrients and micronutrients), genetics, and the environment. Developmental status was assessed in the present study via the ASQ-3 for children aged 1–66 months, with a sensitivity of 88% and specificity of 94% [9]. The ASQ-3 is a suitable developmental screening option in low- to middle-income countries [41]. The developmental assessment revealed that short stature was associated with abnormal problem solving in our study cohort, which is in line with research by Juwita et al., who reported abnormal problem solving as well as personal social and communication skills [24]. There are contradictory reports on the association between vitamin D and development. The present study revealed a relationship between vitamin D and child development, especially in terms of gross motor skills and personal social development. Vitamin D receptors are distributed throughout the brain and vitamin D plays an important role in the development of nerve cells in early life. Yates et al. reported that vitamin D deficiency in pregnant women causes problems with learning and memory [42]. Low vitamin D levels in experimental animals are associated with schizophrenia, especially in the prefrontal cortex [43]. In addition to being known as a high-level brain association center, the frontal area also plays a role in gross motor tasks. The frontal cortex area has four gyri, one of which is the precentral gyrus, which consists of the primary motor cortex responsible for gross motor movements [44]. However, the present results are different from those of research in India, which reported no differences in growth or development in vitamin D-deficient children [45].

This study has several limitations. The neurotrophic factor levels were evaluated in stored biological samples collected from children under the age of 2 years; thus, these levels may not yet have increased, with the blood sampling not taking into account the circadian cycle. Additionally, some factors that may influence development, such as genetics, were not examined.

## 5. Conclusions

Low vitamin D levels are associated with short stature and developmental status, particularly problem solving, gross motor skills, and personal social development in children <2 years of age in the Bandung region of Indonesia. It is recommended that children’s development is monitored from birth, especially up to the first 1000 days of life, via an appropriate tool such as the ASQ-3. Mothers should also be educated on how to prevent deficient vitamin D levels in their children by consuming a balanced diet rich in vitamin D and engaging in outdoor activities to increase sun exposure.

## Figures and Tables

**Table 1:** Characteristics of the research subjects.

Characteristics	Body Length		*p*-Value *
	Short (*n* = 35)	Normal (*n* = 50)	
Gender:			0.038
Male	26 (74.3)	26 (52.0)	
Female	9 (25.7)	24 (48.0)	
Mother’s education:			0.024
Elementary school	12 (34.3)	11 (22.0)	
Junior high school	19 (54.3)	20 (40.0)	
≥high school	4 (11.4)	19 (38.0)	
Mother’s employment:			0.687
Employed	3 (8.6)	3 (6.0)	
Not employed	32 (91.4)	47 (94.0)	
Infant feeding:			0.567
Breastfed	31 (88.6)	43 (86.0)	
Formula	3 (8.6)	3 (6.0)	
Breast milk + formula	1 (2.9)	4 (8.0)	
Income:			0.663
Below UMR	28 (80.0)	38 (76.0)	
Above the UMR	7 (20.0)	12 (24.0)	
Nutritional status:			0.153
Malnutrition	9 (25.7)	5 (10.0)	
Good nutrition	22 (62.9)	37 (74.0)	
Overnutrition	4 (11.4)	8 (16.0)	
Parenting pattern for 2 years:			0.251
According to the KIA book	22 (66.7)	27 (54.0)	
Not according to the KIA book	11 (33.3)	23 (46.0)	

* Based on the chi-square test or Fisher’s exact test.

**Table 2:** Multivariable analysis of factors associated with short stature in children aged <2 years.

Variable	Coef B	SE (B)	*p*-Value	ORadj (IK 95%)
Vitamin D levels ≤ 32.7	1872	0.623	0.003	6.50 (1.92–22.06)
GDNF levels ≤ 12.99	1515	0.641	0.018	4.55 (1.29–15.99)
Male gender	1339	0.571	0.019	3.82 (1.25–11.68)
Mother’s education:				
Elementary school	1719	0.797	0.031	5.58 (1.17–26.62)
Junior high school	1805	0.749	0.016	6.08 (1.40–26.38)

Accuracy = 76.5%; R^2^ (Nagelkerke) = 0.392.

**Table 3:** Comparison in vitamin D content and developmental status in children < 2 years with short and normal stature.

Variable	Body Length		*p*-Value *
	Short (*n* = 35)	Normal (*n* = 50)	
Vitamin D (ng/mL)	27.65 (6.77)	31.89 (10.66)	0.041
2. NGF (ng/mL)	1333.0 (353.62–1606.5)	1249.75 (393.2–1397.9)	0.353
3. GDNF (ng/mL)	15.93 (7.00)	17.63 (5.57)	0.217
4. BDNF (ng/mL)	1723.25 (588.65)	1873.51 (510.67)	0.214
5. Child Development Status (ASQ-3)			*p*-value ^+^
- Communication			0.634
Abnormal	7 (20.0%)	8 (16.0%)	
Normal	28 (80.0%)	42 (84.0%)	
- Gross motor skills			0.219
Abnormal	7 (20.0%)	5 (10.0%)	
Normal	28 (80.0%)	45 (90.0%)	
- Fine motor skills			0.490
Abnormal	13 (37.1%)	15 (30.0%)	
Normal	22 (62.9%)	35 (70.0%)	
- Problem-solving			0.005
Abnormal	11 (31.4%)	4 (8.0%)	
Normal	24 (68.6%)	46 (92.0%)	
- Social personal development			0.523
Abnormal	15 (42.9%)	18 (36.0%)	
Normal	20 (57.1%)	32 (64.0%)	

The data for the vitamin D, GDNF, and BDNF levels are presented as the means ± SDs. For NGF levels, the data are presented as median and interquartile range. * Unpaired *t*-test, except Mann–Whitney test for NGF. ^+^ Based on the chi-square test.

**Table 4:** Relationships between biomarkers and developmental status according to the ASQ-3 in children aged 2–4 years.

Variable	Group					*p*-Value
	I (*n* = 21)		II (*n* = 14)	III (*n* = 31)	IV (*n* = 19)	
Vitamin D, NGF, BDNF, and GDNF levels and development status						
Vitamin D	29.3		27.3	34.1	30.5	0.164
	(24.35–32.5)		(20.9–33.12)	(22.7–37.8)	(27.0–36.8)	
NGF	1276.3		1354.2	1250.2	1242.4	0.693
	(306.85–1591.05)		(763.52–1616.32)	(621.5–1431.8)	(363.3–1375.2)	
GDNF	14.58		15.65	16.4	16.73	0.551
	(10.86–21.50)		(10.53–20.16)	(12.88–21.60)	(14.95–20.20)	
BDNF	1736.68		1703.10	1871.29	1877.13	0.668
	(619.28)		(561.70)	(497.26)	(545.70)	
Vitamin D, NGF, BDNF, and GDNF levels and communication in children with a history of short stature						
Vitamin D		32.0	27.3	28.9	32.8	0.116
		(27.8–32.7)	(23.28–32.45)	(22.22–34.82)	(25.92–37.9)	
NGF		1424.6	1304.65	996.9	1270.25	0.628
		(234.2–1606.5)	(364.69–1584.98)	(172.98–1357.3)	(430.8–1417.4)	
GDNF		13.9	16.44	18.20	17.52	0.472
		(4.62)	(7.45)	(4.57)	(5.78)	
BDNF		1612.56	1750.92	1836.57	1880.54	0.588
		(577.83)	(598.48)	(475.96)	(522.19)	
Vitamin D, NGF, BDNF, and GDNF levels and gross motor skills in children with a history of short stature						
Vitamin D		25.8	28.95	28.4	33.2	0.035
		(14.4–29.3)	(24.0–32.68)	(23.05–29.7)	(22.95–38.0)	
NGF		1237.2	1342.65	1242.1	1266.4	0.770
		(298.8–1606.5)	(364.69–1604.7)	(723.05–1281.75)	(378.75–1422.2)	
GDNF		15.51	16.04	14.94	17.93	0.461
		(9.30)	(6.51)	(6.88)	(5.42)	
BDNF		1602.73	1753.38	1702.85	1892.47	0.477
		(534.28)	(606.80)	(500.78)	(513.76)	
Vitamin D, NGF, BDNF, and GDNF levels and fine motor skills in children with a history of short stature						
Vitamin D		29.03	26.83	30.01	32.69	0.141
		(6.11)	(7.14)	(10.86)	(10.62)	
NGF		1276.3	1342.65	1274.1	1242.1	0.559
		(326.21–1666.95)	(377.15–1526.12)	(1056.6–1431.8)	(363.3–1393.0)	
GDNF		16.69	15.48	17.13	17.84	0.584
		(8.60)	(6.04)	(5.25)	(5.77)	
BDNF		1689.1	1743.48	1938.42	1845.69	0.592
		(578.45)	(607.17)	(533.58)	(505.92)	
Vitamin D, NGF, BDNF, and GDNF levels and personal social development in children with a history of short stature						
Vitamin D		28.8	28.95	34.85	30.35	0.038
		(23.8–32.7)	(23.48–32.52)	(28.1–41.95)	(21.78–34.85)	
NGF		1333.0	1314.3	1278.15	1249.75	0.741
		(298.8–1606.5)	(432.3–1558.98)	(623.02–1570.45)	(390.68–1372.05)	
GDNF		15.93	15.93	16.49	18.27	0.486
		(6.95)	(7.21)	(6.13)	(5.22)	
BDNF		1733.12	1715.85	1788.86	1921.12	0.530
		(577.92)	(612.81)	(557.60)	(484.99)	

Data are presented as the mean and standard deviation (SD) if the data were normally distributed and as the median and interquartile range if the data were not normally distributed. Group I: short stature and abnormal developmental status. Group II: short stature and normal development status. Group III: normal stature and abnormal development status. Group IV: normal stature and normal development status.

**Table 5:** Relationships between vitamin D levels and NGF, BDNF, and GDNF in children < 2 years with a history of short and normal stature.

Correlation	Short Stature		Normal Stature		Combined	
	r	*p*-Value	r	*p*-Value	r	*p*-Value
Vitamin D with NGF	−0.041	0.816 *	−0.015	0.916 *	−0.062	0.574 *
Vitamin D with GDNF	0.018	0.917	0.010	0.946	0.042	0.704
Vitamin D with BDNF	−0.009	0.957	0.097	0.504	0.087	0.429
NGF with GDNF	−0.030	0.863 *	0.041	0.778 *	0.009	0.933 *
NGF with BDNF	−0.031	0.859 *	0.066	0.649 *	0.017	0.876 *
GDNF with BDNF	0.868	<0.001	0.882	<0.001	0.876	<0.001

r = Pearson correlation; * Spearman rank correlation.

**Table 6:** Vitamin D, NGF, BDNF, and GDNF cutoff values for the prediction of short stature in children aged <2 years.

Variable	Short vs. Normal Stature	
	Cutoff Value	AUROC (CI 95%)
Vitamin D (ng/mL)	≤32.7	0.641 (0.530–0.742)
NGF (ng/mL)	>1315.5	0.559 (0.448–0.667)
GDNF (ng/mL)	≤12.99	0.591 (0.479–0.696)
BDNF (ng/mL)	≤1803.6	0.586 (0.474–0.692)

AUROC = area under the receiver operating characteristic.

## Data Availability

The original data used to support the findings of this study are available upon request from the corresponding author due to ethical reasons.
